# PI3Ks Maintain the Structural Integrity of T-Tubules in Cardiac Myocytes

**DOI:** 10.1371/journal.pone.0024404

**Published:** 2011-09-02

**Authors:** Chia-Yen C. Wu, Zhiheng Jia, Wei Wang, Lisa M. Ballou, Ya-Ping Jiang, Biyi Chen, Richard T. Mathias, Ira S. Cohen, Long-Sheng Song, Emilia Entcheva, Richard Z. Lin

**Affiliations:** 1 Department of Physiology and Biophysics and Institute of Molecular Cardiology, Stony Brook University, Stony Brook, New York, United States of America; 2 Department of Biomedical Engineering, Stony Brook University, Stony Brook, New York, United States of America; 3 Department of Medicine, Stony Brook University, Stony Brook, New York, United States of America; 4 Department of Veterans Affairs Medical Center, Northport, New York, United States of America; 5 Division of Cardiovascular Medicine, Department of Internal Medicine, University of Iowa Carver College of Medicine, Iowa City, Iowa, United States of America; 6 Department of Molecular Physiology and Biophysics, Baylor College of Medicine, Houston, Texas, United States of America; The University of Texas at Austin, United States of America

## Abstract

**Background:**

Phosphoinositide 3-kinases (PI3Ks) regulate numerous physiological processes including some aspects of cardiac function. Although regulation of cardiac contraction by individual PI3K isoforms has been studied, little is known about the cardiac consequences of downregulating multiple PI3Ks concurrently.

**Methods and Results:**

Genetic ablation of both p110α and p110β in cardiac myocytes throughout development or in adult mice caused heart failure and death. Ventricular myocytes from double knockout animals showed transverse tubule (T-tubule) loss and disorganization, misalignment of L-type Ca^2+^ channels in the T-tubules with ryanodine receptors in the sarcoplasmic reticulum, and reduced Ca^2+^ transients and contractility. Junctophilin-2, which is thought to tether T-tubules to the sarcoplasmic reticulum, was mislocalized in the double PI3K-null myocytes without a change in expression level.

**Conclusions:**

PI3K p110α and p110β are required to maintain the organized network of T-tubules that is vital for efficient Ca^2+^-induced Ca^2+^ release and ventricular contraction. PI3Ks maintain T-tubule organization by regulating junctophilin-2 localization. These results could have important medical implications because several PI3K inhibitors that target both isoforms are being used to treat cancer patients in clinical trials.

## Introduction

Efficient cardiac mechanical function depends on tight coupling between the electrically excitable myocyte surface membrane and intracellular Ca^2+^ stores in the sarcoplasmic reticulum (SR) [1]. In mature mammalian ventricular myocytes, rapid and synchronous Ca^2+^ release throughout the cell in response to an action potential is made possible by a dense network of plasma membrane invaginations called transverse tubules (T-tubules) [2]. The surface membrane invaginations form near the sarcomere Z lines at regular ∼2 µm intervals along the longitudinal axis of the myocyte. T-tubules are closely juxtaposed (∼12 nm distance) to regions of the SR called junctional domains (jSR) where Ca^2+^ release takes place [3]. Several Ca^2+^-handling proteins are concentrated in these junctional membrane complexes (dyad junctions). Voltage-dependent L-type Ca^2+^ channels (Ca_V_1.2) are located in the T-tubules, and the jSR membrane contains ryanodine receptor 2 (RyR) Ca^2+^ release channels. Propagation of the action potential into the cell interior via the T-tubules activates a relatively small inward Ca^2+^ current (I_Ca,L_) through Ca_V_1.2. This Ca^2+^ rapidly diffuses across the narrow dyadic cleft to induce a much larger release of Ca^2+^ from the SR through RyR, leading to myocyte contraction. The density of T-tubules, their juxtaposition to the jSR, and proper localization to the contractile machinery are necessary for efficient Ca^2+^-induced Ca^2+^ release and synchronized contraction in ventricular myocytes [4], [5].

T-tubules in mammalian ventricular myocytes develop after birth [6] and can undergo remodeling or loss in heart disease [4], [7]–[10]. Although the exact mechanisms that control T-tubule formation and maintenance are unknown, junctophilin-2 (JP-2) has been implicated in regulating this cellular structure. The four members of the junctophilin family are believed to tether the plasma membrane to the SR/endoplasmic reticulum in excitable cells [11], [12]. JP-2, the major junctophilin isoform expressed in the heart, has a transmembrane domain at the carboxyl terminus that anchors the protein in the SR membrane and eight *m*embrane *o*ccupation and *r*ecognition *n*exus (MORN) motifs in the amino terminus that are thought to interact with the plasma membrane to stabilize the junctional membrane complexes [11], [13]. Knockdown of JP-2 in cultured adult mouse cardiac myocytes disrupted the T-tubule network [5]. In addition, knockout or knockdown of JP-2 in the mouse heart led to T-tubule disorganization, reduced number and altered morphology of junctional membrane complexes, and abnormal Ca^2+^ transients [11], [14]. Currently, it is unknown if the function of JP-2 is regulated by cell signaling pathways.

Class IA phosphoinositide 3-kinases (PI3Ks) are activated by various hormones and phosphorylate the membrane lipid phosphatidylinositol (4,5)-bisphosphate to produce the second messenger phosphatidylinositol (3,4,5)-trisphosphate (PIP_3_) [15]. The enzymes consist of a catalytic subunit (p110α, p110β, or p110δ) associated with various regulatory subunits collectively referred to as p85. We previously showed that cardiac-specific ablation of p110α, but not p110β, resulted in reduced Ca^2+^ transients and a mild contractile defect in adult mice [16]. In this study, we used chronic and acute models of PI3K deletion to study the effects of ablating p110α, p110β or both PI3Ks on T-tubule structure. We show that simultaneous loss of both p110α and p110β throughout development or in adulthood disrupts the localization of JP-2 and the integrity of the T-tubule network, leading to severely compromised myocyte contraction and fatal heart failure.

## Materials and Methods

### Materials

Ca_V_1.2 antibody was from Alomone Labs. RyR antibody was from Affinity Bioreagents. Di-8-ANEPPS, FM 4-64, JP-2 antibody for immunostaining, goat anti-rabbit antibody conjugated to Alexa Fluor 488 and goat anti-mouse antibody conjugated to Alexa Fluor 647 were from Invitrogen. p110α, p110β, and phospho-specific Akt antibodies were from Cell Signaling Technology. Pan-p85 antibody was from Millipore; antibodies for Akt and JP-2 (for western blotting) were from Santa Cruz Biotechnology. Antibody to GAPDH was from Sigma-Aldrich and to caveolin-3 was from BD Transduction Laboratories.

### Animals

All animal-related experimental protocols for this study were approved by the Stony Brook University Institutional Animal Care and Use Committee under the approval ID #1712. To minimize animal suffering, mice exhibiting signs of distress such as inability to keep upright or ruffled fur were euthanized. All genetically modified mice were in a mixed C57BL/6 and 129 genetic background. For the chronic model of PI3K ablation, we used a breeding program between p110α^Flox/WT^ and p110β^Flox/WT^ mice [16] and muscle creatine kinase (MCK)-Cre mice [17]. Offspring from the last breeding step had 16 possible genotypes; in this study, we used only p110α^Flox/Flox^;p110β^WT/WT^;MCK-Cre (called α^−/−^), p110α^WT/WT^;p110β^Flox/Flox^;MCK-Cre (called β^−/−^) and p110α^Flox/Flox^;p110β^Flox/Flox^;MCK-Cre (called dKO) mice, and siblings that did not express MCK-Cre served as controls. Mice with chronic deletion of PI3K were studied at 5 weeks of age or, where explicitly stated, at 3 weeks of age. For the acute model of PI3K ablation, p110α^Flox/Flox^ and p110β^Flox/Flox^ mice were bred to transgenic mice expressing α myosin heavy chain (αMHC)-MerCreMer [18] to generate MerCreMer;p110α^Flox/Flox^;p110β^Flox/Flox^ double knockout mice (called ind-dKO) and p110α^Flox/Flox^;p110β^Flox/Flox^ littermates without MerCreMer (called ind-control) that served as controls. Ind-control and ind-dKO mice older than 8 weeks of age were injected with tamoxifen (60 mg/kg) for 5–7 days to induce deletion of the PI3K genes.

### Echocardiography

Mice were anesthetized with 1–2% isoflurane and transthoracic echocardiography was performed as previously described [19]. Left ventricular end-diastolic diameter (LVEDD), left ventricular end-systolic diameter (LVESD), septal wall thickness and posterior wall thickness were obtained from 9 cardiac cycles for each animal. Fractional shortening was calculated as (LVEDD – LVESD)/LVEDD×100.

### T-tubule visualization *in vitro* and immunofluorescence microscopy

Cardiac ventricular myocytes were isolated as described earlier [16]. T-tubules were visualized by incubating living myocytes with 10 µM di-8-ANEPPS in KB solution (5 mM Hepes, 5 mM MgSO_4_, 10 mM glucose, 90 mM KCl, 30 mM K_2_HPO_4_, 5 mM sodium pyruvate, 1.09 mM NaOH, 0.5 mM EGTA, 20 mM taurine, 5 mM creatine, and 5 mM sodium butyrate, pH 7.3) for 10 min, followed by a 10 min washout in KB solution. Di-8-ANEPPS was excited at 488 nm and emission intensity was measured at 506 nm. Images were acquired using an Olympus FluoView^TM^ FV1000 Confocal Microscope with 60X oil lens (NA = 1.42) at room temperature. The acquisition software was Olympus FluoView^TM^ FV1000 (Version 1.7). For immunofluorescence microscopy, isolated myocytes were fixed for 10 min in 3.7% formaldehyde, washed thoroughly in phosphate-buffered saline and permeabilized using 0.2% Triton X-100 with 5% fetal bovine serum (FBS) for 5 min. After washing in 1% FBS solution, cells were labeled with primary antibodies overnight at 4°C. The next day, the cells were washed with 1% FBS solution and then incubated with Alexa Fluor-labeled secondary antibodies for 1 h. The cells were rinsed with phosphate-buffered saline and then mounted in VectaShield (with DAPI) (Vector Laboratories) for imaging using the Olympus confocal system.

For spatial spectrum analysis, regions of interest without nuclei were first selected. After background subtraction (to avoid staining variation and noise), power spectrum was computed by 1D fast Fourier transform along the longitudinal dimension for each sample, averaging multiple lines (>10). Normalized power (calculated as peak power divided by mean power from 0.2 to 0.4 µm^−1^ for each cell) was averaged and used to compare groups of cells. Colocalization analysis was done using commercial software from Imaris (Bitplane Inc.). An unbiased automatic thresholding method [20] was applied before calculating Pearson's coefficients and colocalization percentages.

### T-tubule visualization *in situ*

*In situ* T-tubule imaging and 2D Fourier analysis were performed as previously described [5]. T-tubules in isolated hearts were stained with either FM 4–64 or di-8-ANEPPS during Langendorff perfusion and imaged by confocal microscopy [5].

### Sarcomere length and Ca^2+^ transient measurements

Isolated myocytes were electrically stimulated at a frequency of 1 Hz. Contractile properties were assessed using a video-based sarcomere spacing acquisition system and Fourier-based analysis software (IonOptix) as previously described [16]. For Ca^2+^ transients, myocytes loaded with Fluo-4 AM were excited at 480 nm and emission intensity of Fluo-4 at 535 nm was measured using an IonOptix photometry system as previously described [16]. Fluorescence values (F) were normalized to each tracing's own minimum fluorescence (F_Diastolic_) to correct for differences in Fluo-4 loading. Ca^2+^ transient amplitude was calculated as the difference between peak F/F_Diastolic_ and trough F/F_Diastolic_.

### Insulin treatment

Mice fasted overnight were anesthetized with 100 mg/kg body weight ketamine and 10 mg/kg body weight xylazine. The inferior vena cava was exposed by laparotomy through a midline incision. Human insulin (Novolin R, Novo Nordisk) at 5 U/kg body weight or an equal volume of saline was injected through the inferior vena cava. Hearts were collected 5 min later and stored in liquid nitrogen until use.

### Sample preparation and western blotting

Mouse ventricular microsomes were prepared as previously described [21]. Mouse heart lysates were prepared in RIPA buffer (50 mM Hepes, pH 7.5, 50 mM NaCl, 50 mM NaF, 10 mM sodium pyrophosphate, 5 mM EDTA, 1 mM NaVO_4_, 0.25% sodium deoxycholate, 1% NP-40, and protease inhibitor cocktail (Sigma-Aldrich)). Cardiac myocytes were isolated and cell lysates were prepared as previously described [16]. After immunoblotting, signals were visualized using an ECL kit (PerkinElmer Life Sciences), CSPD chemiluminescence kit (Applied Biosystems) or the Odyssey Infrared Imaging System (LI-COR Biosciences).

### Heart histology

Hearts were fixed by perfusion with 10% buffered formalin and embedded in paraffin. Sections of 10 µm thickness were cut and stained with hematoxylin and eosin. Images were acquired using the Olympus IX 70 Fluorescence Microscope with 20X lens or Olympus (BX41) Microscope with 20X lens. The acquisition software was Olympus DP Controller for the Olympus IX 70 microscope and CellSens Dimension Imaging for the Olympus (BX41) microscope.

### Real-time RT-PCR

RNA was isolated from hearts of 36–37-day-old mice using Tri Reagent (Sigma-Aldrich). RNA was converted to cDNA using the iScript cDNA Synthesis Kit (Bio-Rad) and analyzed by real-time PCR using the SYBR Green PCR Kit (Qiagen) with a DNA Engine Opticon (MJ Research). Results were quantified using a previously described method [22]. Oligonucleotide primers used were: βMHC—TGCAAAGGCTCCAGGTCTGAGGGC and GCCAACACCAACCTGTCCAAGTTC. Results were normalized to the expression of 18S RNA.

### Statistics

Numerical data are presented as mean ± SEM or median ± range. Comparison between groups was evaluated using one-way analysis of variance (ANOVA) with *post-hoc* Fisher least significant difference test. Comparison between two groups was evaluated using Student's *t*-test or Mann-Whitney U test. Results were considered significant if the *P* value was <0.05.

## Results

### Chronic and acute mouse models of PI3K deletion

To investigate the impact of reduced PI3K activity on the heart, we created chronic and acute mouse models of p110α and/or p110β deletion. The chronic model used a breeding program between p110α^Flox/WT^ and p110β^Flox/WT^ mice and animals carrying a Cre recombinase transgene under the control of the MCK promoter to generate p110α or p110β knockout mice (α^−/−^ and β^−/−^, respectively) and double p110α/p110β knockout mice (dKO). Siblings that did not express Cre served as controls. The acute model employed a tamoxifen-inducible system previously used by us to create single PI3K knockout mice [16]. For the present study, mice homozygous for floxed p110 alleles were bred to transgenic mice expressing tamoxifen-activated Cre (MerCreMer) under the control of the αMHC promoter to generate double p110α/p110β knockout mice (ind-dKO) and littermates without MerCreMer to serve as controls (ind-control). Ind-control and ind-dKO mice were analyzed after injection with tamoxifen to induce deletion of the PI3K genes.

Western blotting showed that the targeted p110α and/or p110β proteins were appropriately down-regulated in the single and double knockout myocytes in both models (Fig. S1a,c). Chronic deletion of p110α also appeared to result in decreases in the p110β and p85 proteins, and a larger decrease in p85 was seen in dKO myocytes (Fig. S1a). A compensatory decrease in p85 was not consistently seen after acute deletion of p110α and p110β (Fig. S1c). To evaluate the effect of gene ablation on PI3K signaling, we examined the phosphorylation of Akt in hearts of control and knockout animals after injection with insulin. In the chronic deletion model, insulin-induced Akt phosphorylation was decreased in α^−/−^ hearts and further reduced in dKO hearts, but there was little or no change in β^−/−^ hearts as compared to the control (Fig. S1b). Interestingly, the total amount of Akt protein was increased in both dKO and α^−/−^ hearts (Fig. S1b). Insulin-induced Akt phosphorylation was also blunted in the heart of ind-dKO mice (Fig. S1d).

### Cardiac double PI3K knockout mice develop decompensated heart failure

**Figure 1:**
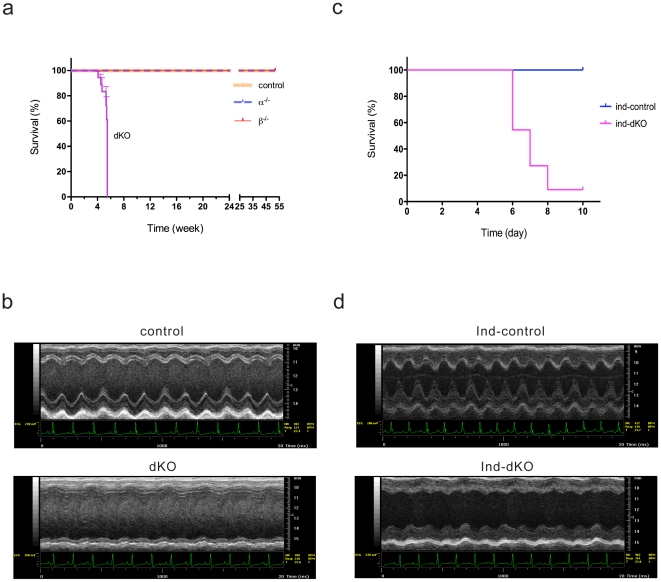
Ablation of both p110α and p110β in cardiac myocytes causes early death in mice. (**a**) Kaplan-Meier survival curves for control, α^−/−^, β^−/−^ and double knockout (dKO) mice (*n* = 18 for dKO and *n* = 20 for the other groups; *P<0.0001 as compared to control, Mantel-Cox test). (**b**) Representative M-mode transthoracic views and electrocardiograms of control and dKO hearts. (**c**) Kaplan-Meier survival curves for inducible dKO (ind-dKO) mice and their controls (ind-control) (*n* = 11 per group; *P<0.0001, Mantel-Cox test). (**d**) Representative M-mode transthoracic views and electrocardiograms of ind-dKO and ind-control hearts.

At birth, all four groups of mice in the chronic PI3K deletion model behaved normally and were indistinguishable by physical appearance. However, the dKO mice became less active one or two days before death and all dKO mice died before 40 days of age (Fig. 1a). There was no significant difference in the mortality rate of α^−/−^ or β^−/−^ mice as compared to control animals (Fig. 1a). Both left and right ventricles were dilated in the dKO hearts, whereas no obvious gross changes were observed in the single PI3K knockout hearts (Fig. S2a). The mean heart weight (HW) to body weight (BW) ratio was increased by 42% in dKO mice as compared to controls (10.76±0.22 mg/g *vs.* 7.61±0.09 mg/g, *n* = 3 per group, *P* = 0.0003, *t*-test). Higher magnification views of hematoxylin and eosin-stained heart sections did not reveal any obvious differences in organ structure in the three groups of chronic knockout mice, indicating that the early mortality of dKO mice was not due to gross defects in cardiac architecture (Fig. S2b). No localized areas of necrosis or myocyte damage were detected in the dKO sections (Fig. S2b).

We next used echocardiography to compare the cardiac function of 5-week-old dKO and control animals. As shown in Fig. 1b, the dKO mouse exhibited very weak cardiac contractions even though the electrocardiogram showed a normal sinus rhythm. Representative echocardiograms of control (Video S1) and dKO (Video S2) mice are provided in the Supporting Information. Echocardiographic measurements showed that average left ventricular end-diastolic diameter, left ventricular end-systolic diameter and posterior wall thickness were significantly altered in dKO hearts (Table 1). In addition, the calculated average fractional shortening was dramatically decreased by 70% in dKO hearts as compared to controls (Table 1). Consistent with the echocardiography findings, we found markedly increased βMHC mRNA levels, which are often associated with cardiac dysfunction, in dKO hearts as compared to single knockouts or controls (Fig. S2c). This severely compromised heart function is most likely the cause of death in the chronic dKO mice.

**Table 1:** Echocardiographic measurements.

	Control	dKO (5 wk)	dKO (3 wk)	Ind-control	Ind-dKO
HR (bpm)	430 ± 20	327 ± 13		361 ± 27	369 ± 8
LVEDD (mm)	3.25 ± 0.09	*3.96 ± 0.15	*2.90 ± 0.12	3.85 ± 0.18	4.38 ± 0.26
LVESD (mm)	1.65 ± 0.19	*3.38 ± 0.11	1.88 ± 0.11	2.28 ± 0.24	^†^3.80 ± 0.22
SWT (mm)	0.15 ± 0.03	0.13 ± 0.03	0.15 ± 0.02	0.13 ± 0.03	^†^0.35 ± 0.06
PWT (mm)	0.88 ± 0.05	*0.72 ± 0.07	0.61 ± 0.06	0.97 ± 0.07	0.96 ± 0.08
FS (%)	49.4 ± 5.0	*14.7 ± 2.7	35.3 ± 2.72	40.9 ± 4.6	^†^13.6 ± 2.5

LVEDD, left ventricular end-diastolic diameter; LVESD, left ventricular end-systolic diameter; SWT, septal wall thickness; PWT, posterior wall thickness; FS, fractional shortening; HR, heart rate in beats per minute (bpm). Values are mean ± SEM. *Significantly different from the Control group, *t*-test. ^†^Significantly different from the Ind-control group, *t*-test. *n* = 3 per group.

Early death due to chronic deletion of p110α and p110β could be due to developmental abnormalities in cardiac myocytes that lead to heart failure. We used the acute model of PI3K deletion to determine whether adult animals are protected from the deleterious effects of PI3K ablation in the heart. In the absence of tamoxifen injection, adult ind-control and ind-dKO mice were indistinguishable by physical appearance and behavior, and mortality was the same in the two groups for up to 1 year. However, ablation of both PI3Ks upon tamoxifen injection of ind-dKO mice rapidly led to death (Fig. 1c). Tamoxifen injections did not cause early mortality in ind-control mice (Fig. 1c). After tamoxifen treatment for 4–5 days, ind-dKO mice lost weight (−1.1±0.3 g *vs.* +1.5±0.2 g in ind-controls; *n* = 7 per group, *P* = 8.3×10^−6^, *t*-test) and moved less. Echocardiography showed that the cardiac function of ind-dKO mice was also markedly compromised (Fig. 1d and Table 1). The mean HW/BW ratio was increased by 59% in ind-dKO mice as compared to ind-controls (11.72±0.33 mg/g *vs.* 7.87±0.13 mg/g, *n* = 3–4 per group, *P* = 0.0027, *t*-test). Histological examination of ind-dKO hearts after tamoxifen treatment showed that both ventricles were severely dilated, but no localized areas of necrosis or myocyte damage were observed (Fig. S2d,e). These results indicate that loss of both p110α and p110β either throughout development or in adulthood severely compromises the contractile function of the heart.

### Reduced Ca^2+^ transients and contractility

**Figure 2:**
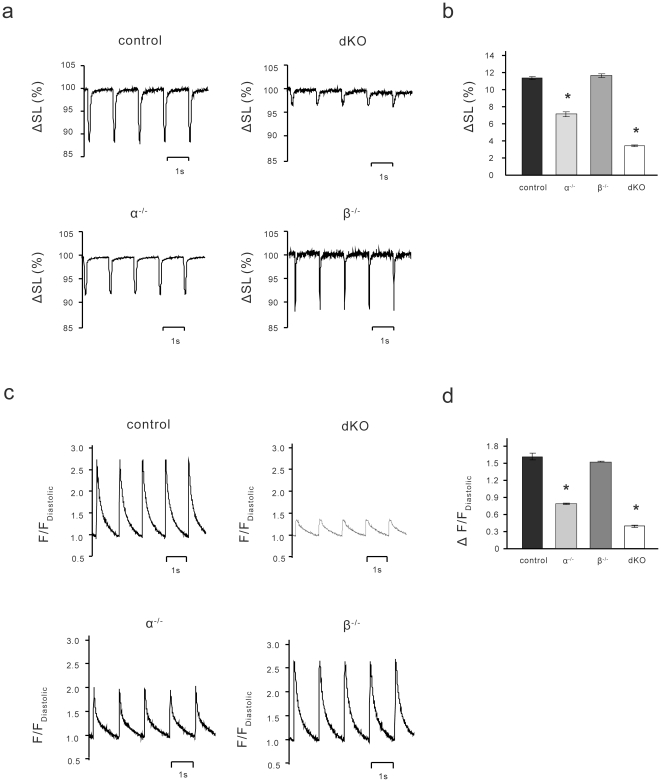
dKO myocytes have defective contractile function and Ca^2+^ transients. (**a**) Representative contractility tracings measuring % change in sarcomere length (ΔSL%) for control, α^−/−^, β^−/−^ and dKO myocytes. (**b**) Summary data of contractility in control (*n* = 25), α^−/−^ (*n* = 10), β^−/−^ (*n* = 14) and dKO (*n* = 22) myocytes. (**c**) Representative Ca^2+^ transient tracings from control, α^−/−^, β^−/−^ and dKO myocytes. Ca^2+^-associated fluorescence (F) was normalized to each tracing's own minimum fluorescence (F_Diastolic_). (**d**) Summary data of Ca^2+^ transient amplitudes from control (*n* = 13), α^−/−^ (*n* = 10), β^−/−^ (*n* = 6) and dKO (*n* = 17) myocytes. Values in b and d are mean ± SEM (*P<0.01 as compared to control, one-way ANOVA with *post-hoc* Fisher's test).

To investigate potential mechanisms that could cause heart failure in the dKO mice, we studied contractility and Ca^2+^ handling in ventricular myocytes isolated from 35-day-old mice in the four groups from the chronic PI3K deletion model. Most of the control myocytes contracted robustly when electrically stimulated. However, ∼90% of the dKO myocytes did not visibly contract under the same conditions, and those that did contract did so poorly. Myocyte contractile function was quantified by measuring changes in sarcomere length in visibly contracting cells. Contractility of the dKO myocytes was dramatically reduced by 71% as compared to control cells (Fig. 2a,b). This decrease was comparable to the decrease in fractional shortening calculated from the echocardiography data (Table 1). Contractility was decreased by 38% in α^−/−^ myocytes as compared to controls but unaffected in β^−/−^ myocytes (Fig. 2a,b). We next measured Ca^2+^ transients in ventricular myocytes isolated from all four groups of mice. The dKO myocytes exhibited Ca^2+^ transient amplitudes that were only 25% of control values (Fig. 2c,d). Chronic ablation of p110α alone caused a more moderate decrease in Ca^2+^ transient amplitude to 48% of the control, whereas ablation of p110β had no effect on Ca^2+^ transients (Fig. 2c,d).

### Misalignment of Ca_V_1.2 and RyR

**Figure 3:**
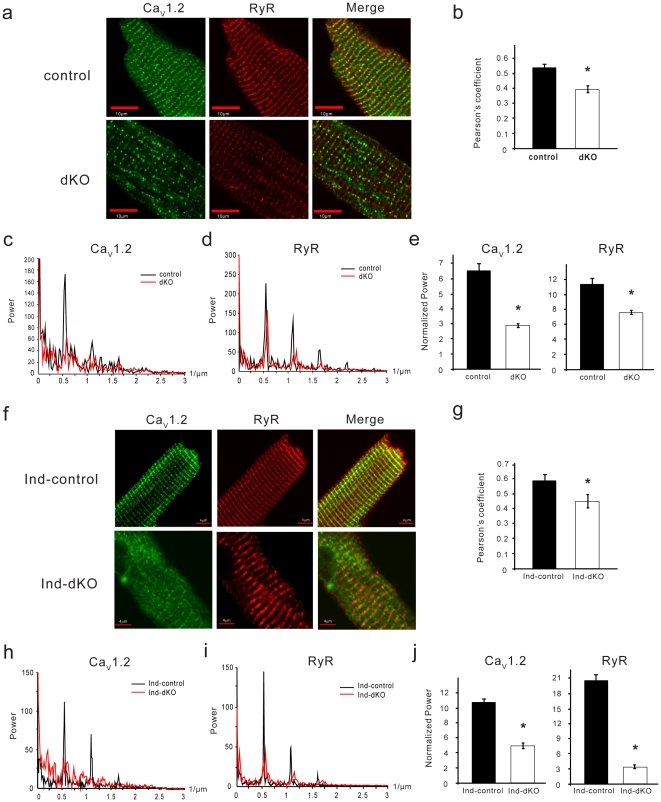
Ca_V_1.2/RyR mismatch in dKO myocytes. (**a**) Confocal microscopy images of myocytes from control and dKO mice colabeled with antibodies against Ca_V_1.2 (green) and RyR (red). (**b**) Pearson's coefficient for colocalization of Ca_V_1.2 and RyR (*n* = 19 cells from ≥3 hearts per group). Data shown are mean ± SEM (*P = 8.1×10^−9^, *t*-test). 1D Fourier analysis of spatial frequency for Ca_V_1.2 (**c**) and RyR (**d**) along the longitudinal axis of representative myocytes (averaged value from >10 lines). (**e**) The normalized output of 1D Fourier transform analysis from 19–20 cells for each group. Data shown are mean ± SEM (*P = 0.0001 (Ca_V_1.2) and 0.015 (RyR), *t*-test). (**f**) Representative ind-dKO and ind-control myocytes colabeled with antibodies against Ca_V_1.2 (green) and RyR (red). (**g**) Pearson's coefficient for colocalization of Ca_V_1.2 and RyR (*n* = 13–16 cells from ≥3 hearts per group). Data shown are mean ± SEM (*P = 0.016, *t*-test). 1D Fourier analysis of spatial frequency for Ca_V_1.2 (**h**) and RyR (**i**) along the longitudinal axis of representative myocytes (averaged value from >10 lines). (**j**) The normalized output of 1D Fourier transform analysis from 19 cells for each group. Data shown are mean ± SEM (*P = 9.8×10^−6^ (Ca_V_1.2) and 4.3×10^−7^ (RyR), *t*-test).

We next asked whether the decrease in Ca^2+^ transient amplitude in dKO myocytes might be due to a change in the relative positioning of Ca_V_1.2 and RyR in the junctional membrane complex. Staining of isolated myocytes from control mice in the chronic PI3K deletion model with antibodies to Ca_V_1.2 (green) and RyR (red) showed that the two proteins colocalized (yellow) in highly organized striations (Fig. 3a). The striated pattern of Ca_V_1.2 and colocalization with RyR were visibly reduced in the dKO cells (Fig. 3a). Pearson's coefficient analysis showed a significant decrease in colocalization of Ca_V_1.2 and RyR in the dKO myocytes (Fig. 3b). In addition, western blotting of heart microsome preparations showed a reduction in the level of both proteins in the dKO samples (Fig. S3a). The degree of spatial periodicity of the two fluorescent signals in stained cells was quantified using 1D Fourier analysis over multiple lines along the longitudinal axis. Periodic spacing of about 2 µm (∼0.5 µm^−1^ dominant spatial frequency with a harmonic at ∼1 µm^−1^) was seen for Ca_V_1.2 and RyR in a representative control cell (Fig. 3c,d). The dominant frequency for the Ca_V_1.2 signal in the dKO cell was almost lost (Fig. 3c), and that for RyR was reduced as compared to the control (Fig. 3d). A normalized output of the Fourier transform revealed significant differences in the spatial organization of Ca_V_1.2 and RyR between control and dKO cells (Fig. 3e). Immunostaining of myocytes from the acute PI3K deletion model also showed reduced colocalization of Ca_V_1.2 and RyR in ind-dKO *vs.* ind-control cells (Fig. 3f,g). Unlike the dKO hearts, a reduction in Ca_V_1.2 protein was not seen in ind-dKO samples (Fig. S3a). 1D Fourier analysis showed a significant loss of spatial organization of Ca_V_1.2 and RyR in the ind-dKO cells (Fig. 3h-j).

### Disrupted T-tubule network

**Figure 4:**
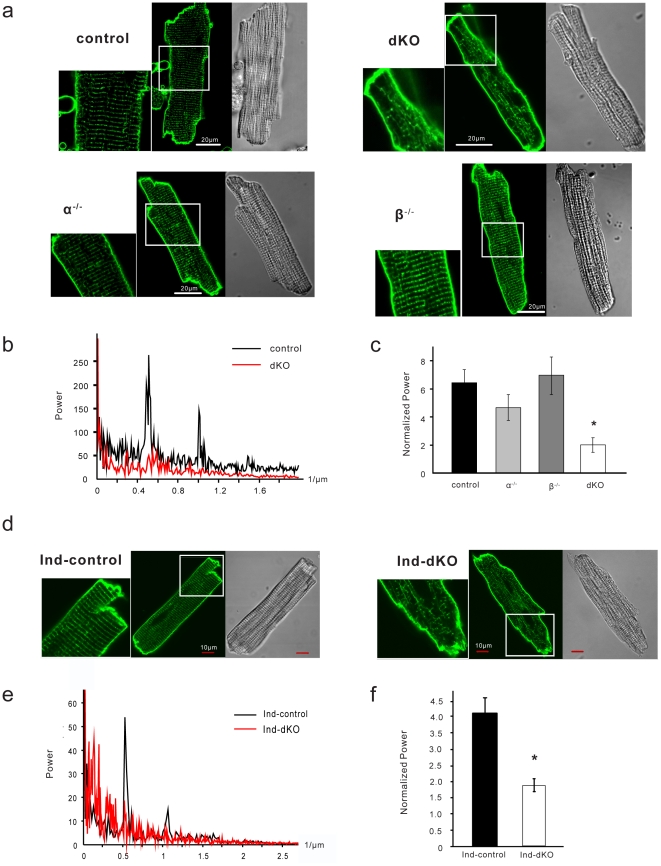
T-tubule disruption in dKO myocytes. (**a**) Confocal microscopy images of myocytes isolated from control, α^−/−^, β^−/−^ and dKO hearts and stained with di-8-ANEPPS (green). The small panels on the left are 2x magnified views of the boxed areas. Phase contrast images of the same cells are displayed on the right. (**b**) 1D Fourier analysis of spatial frequency along the longitudinal axis of representative di-8-ANEPPS–stained control and dKO myocytes (averaged value from >10 lines). (**c**) Normalized power was computed as a measure of T-tubule spatial organization (*n* = 8–20 cells from ≥3 hearts per group). Data shown are mean ± SEM (*P<0.01 as compared to control, one-way ANOVA with *post-hoc* Fisher's test). (**d**) Confocal microscopy images of myocytes isolated from ind-dKO and ind-control hearts and stained with di-8-ANEPPS. Smaller panels are 2x magnified views of the boxed areas. Phase contrast images of the same cells are displayed on the right. (**e**) 1D Fourier analysis of spatial frequency along the longitudinal axis of representative di-8-ANEPPS–stained cells (averaged value from >10 lines). (**f**) Normalized power as a measure of T-tubule spatial organization (*n* = 15–25 cells from ≥3 hearts per group). Data shown are mean ± SEM (*P = 4.8×10^−4^, *t*-test).

Loss of the organized striated pattern for Ca_V_1.2 in dKO and ind-dKO cells suggested that the T-tubule network might be affected by the loss of PI3Ks. To visualize T-tubules, we stained living isolated ventricular myocytes with di-8-ANEPPS, a lipophilic dye that cannot enter the cell and that stains the cell surface membrane. Confocal microscopy images of representative control, α^−/−^ and β^−/−^ myocytes from the chronic deletion model show that the T-tubule network exhibited a highly organized striated pattern (Fig. 4a). In contrast, the T-tubule network in dKO myocytes was severely disrupted and the striated arrangement was almost completely lost (Fig. 4a). 1D Fourier analysis of the di-8-ANEPPS signal showed periodic T-tubule spacing of about 2 µm in a representative control cell (Fig. 4b), commensurate with the sarcomere spacing visible in the phase contrast image (Fig. 4a). There was no discernable dominant frequency in the Fourier spectrum for the dKO cell (Fig. 4b), despite its normal sarcomere appearance in the phase contrast image (Fig. 4a). This result suggests that T-tubule disruption is not due to damage of dKO cells during the isolation procedure. A normalized output of the Fourier transform revealed a significant difference in the spatial organization of T-tubules between the control and dKO groups, while the single knockouts were not significantly different from the control (Fig. 4c). Acute deletion of both p110α and p110β in adult myocytes also led to severe T-tubule disruption as revealed by di-8-ANEPPS staining of ind-control and ind-dKO myocytes (Fig. 4d) and 1D Fourier analysis of the di-8-ANEPPS signals (Fig. 4e,f). We also visualized the T-tubules in isolated fixed cells by immunostaining for caveolin-3, which is localized to the plasma membrane [23]. Consistent with the results obtained with di-8-ANEPPS, caveolin-3 staining of dKO and ind-dKO myocytes showed a disordered pattern as compared to the orderly striations in control and ind-control myocytes (Fig. S3b). The protein level of caveolin-3 was not affected by PI3K deletion in either model (Fig. S3a).

**Figure 5:**
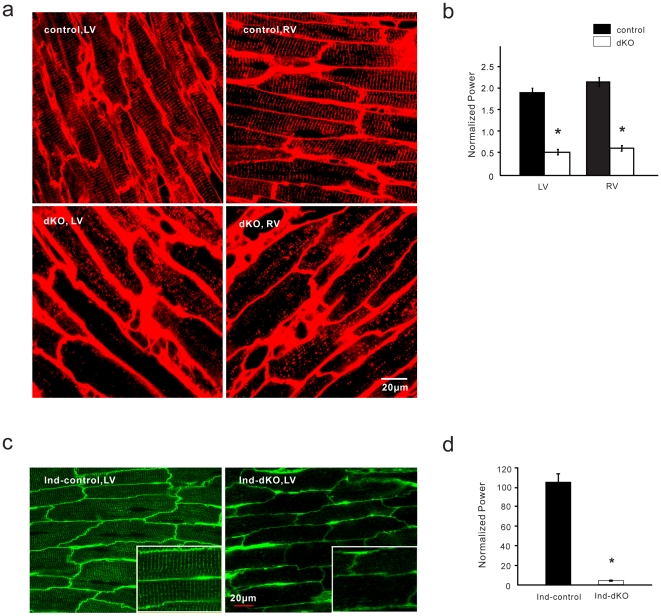
T-tubule disruption in dKO hearts. (**a**) *In situ* T-tubule imaging of myocytes in the left ventricle (LV) and right ventricle (RV) of control and dKO hearts perfused with FM 4–64. (**b**) T-tubule organization was analyzed by 2D Fourier analysis. *n* = 10 images from each group. *P = 8.3×10^−10^ and 6.8×10^−5^ (*t*-test) for LV and RV, respectively. (**c**) *In situ* T-tubule imaging of myocytes in the LV of ind-dKO and ind-control hearts perfused with di-8-ANEPPS. (**d**) T-tubule organization was analyzed by 2D Fourier analysis. Data shown are mean ± SEM from 10 cells (*P = 9.7×10^−4^, *t*-test).

To further study T-tubule organization without the myocyte isolation procedure, we used a confocal imaging technique to visualize T-tubules in epicardial myocytes on the intact heart and analyzed the images using 2D Fourier analysis [5]. Control hearts from 35-day-old mice in the chronic PI3K deletion model showed a mature T-tubule system in both left and right ventricular myocytes (Fig. 5a). However, dKO myocytes in both ventricles displayed severe disruption of the T-tubule network (Fig. 5a,b). In the acute PI3K knockout model, there was marked T-tubule loss and disorganization of the striated arrangement of T-tubules in left ventricular myocytes of ind-dKO hearts (Fig. 5c,d).

**Figure 6:**
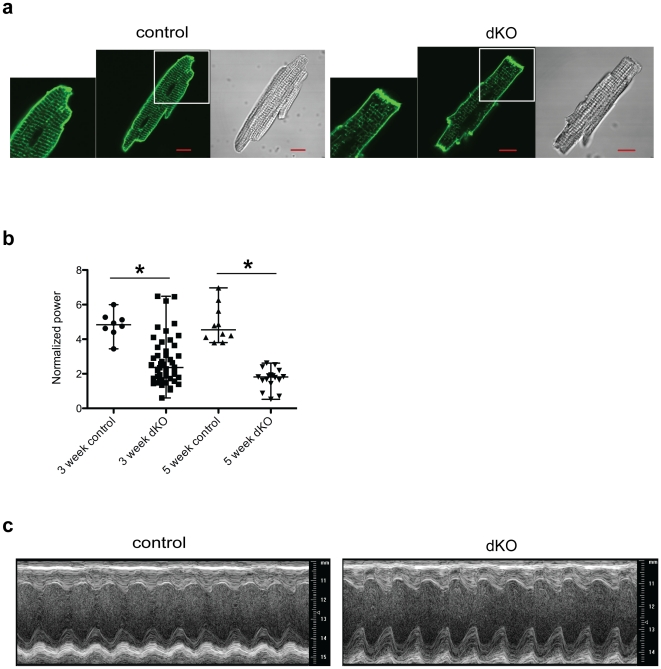
T-tubule abnormality in 3-week-old dKO myocytes. (**a**) Confocal microscopy images of myocytes isolated from 3-week-old control and dKO hearts and stained with di-8-ANEPPS. Phase contrast images of the same cells are displayed on the right. (**b**) Normalized power was computed for each myocyte as a measure of T-tubule spatial organization (from ≥3 hearts per group). Data for individual cells are shown and bars are median, maximum and minimum values (*P<0.01 as compared to control, Mann-Whitney U test). (**c**) Representative M-mode transthoracic echocardiographic views of 3-week-old control and dKO hearts.

To address the question of whether T-tubule disruption is a primary effect of PI3K deletion or a consequence of heart failure, we examined younger dKO mice (3 weeks old) in the chronic knockout model. The T-tubule network in control myocytes was already well developed at this age (Fig. 6a,b). T-tubules in most of the dKO myocytes were disrupted (Fig. 6a,b), even though echocardiography showed that the mice were not in heart failure (Fig. 6c and Table 1). However, in contrast to the 5 week-old dKO myocytes, some of the younger dKO myocytes had normal T-tubule networks, suggesting that formation of these structures does not require PI3K (Fig. 6b). These results suggest that PI3K deletion in the dKO mice causes disruption of the T-tubules, which then leads to heart failure.

Taken together, these results show that PI3Ks are crucial for maintaining the T-tubule network in ventricular myocytes. A defect in T-tubule organization in dKO and ind-dKO myocytes leads to mislocalization of Ca_V_1.2 in relation to RyR, resulting in orphaned RyRs [4]. This abnormal structure contributes to the reduced contractile function of hearts lacking p110α and p110β.

### Altered localization of JP-2

**Figure 7:**
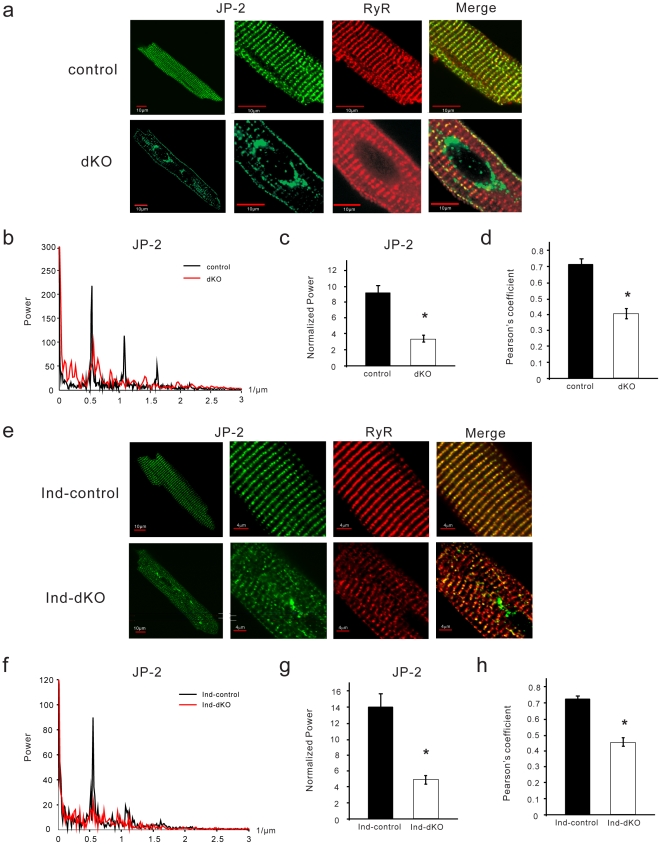
Disrupted JP-2 distribution in dKO myocytes. (**a**) Confocal microscopy images of ventricular myocytes from control and dKO mice colabeled with antibodies against JP-2 (green) and RyR (red). (**b**) 1D Fourier analysis of spatial frequency for JP-2 along the longitudinal axis of representative cells (averaged value from >10 lines). (**c**) Normalized power was calculated as a measure of JP-2 spatial organization (*n* = 15–16 cells from ≥3 hearts per group). Data shown are mean ± SEM (*P = 7.7×10^−9^, *t*-test). (**d**) Pearson's coefficient for colocalization of JP-2 and RyR (*n* = 15–16 cells from ≥3 hearts per group). Data shown are mean ± SEM (*P = 5.8×10^−8^, *t*-test). (**e**) Representative ventricular myocytes from ind-dKO and ind-control mice colabeled with antibodies against JP-2 (green) and RyR (red). (**f**) 1D Fourier analysis of spatial frequency for JP-2 along the longitudinal axis of representative cells (averaged value from >10 lines). (**g**) Normalized power for JP-2 spatial organization (*n* = 24–26 cells from ≥3 hearts per group). Data shown are mean ± SEM (*P = 4.1×10^−5^, *t*-test). (**h**) Pearson's coefficient for colocalization of JP-2 and RyR (*n* = 15–16 cells from ≥3 hearts per group). Data shown are mean ± SEM (*P = 1.0×10^−12^, *t*-test).

JP-2 plays a critical role in the maintenance of T-tubule organization in cardiac myocytes [5], [14]. Western blotting showed that JP-2 levels were not decreased in dKO or ind-dKO hearts as compared to their respective controls (Fig. S3a). We therefore used immunofluorescence microscopy to examine the intracellular localization of JP-2. JP-2 was distributed in an organized striated pattern that overlapped with RyR staining in control cells of the chronic (Fig. 7a–d) and acute (Fig.7e–h) PI3K deletion models. The striated arrangement of JP-2 and colocalization with RyR were significantly reduced in dKO (Fig. 7a–d) and ind-dKO (Fig. 7e–h) myocytes. In addition, dKO myocytes contained clusters of JP-2, especially in the perinuclear region (Fig. 7a). The appearance of perinuclear JP-2 was variable in the ind-dKO cells. These results suggest that p110α and p110β are required for correct localization of JP-2 so that it can properly appose Ca_V_1.2 in the T-tubules to RyR in the jSR to form efficient Ca^2+^ release units.

## Discussion

Experiments in genetically modified mice have shown that PI3Ks regulate physiological heart function in a variety of ways. The class IA PI3K p110α controls heart size and enhances cardiac contractility by increasing I_Ca,L_ in ventricular myocytes [16], [24]–[26]. PI3K p110β positively regulates autophagy in the heart, but how that affects cardiac function is not clear [16], [27]. The class IB PI3K catalytic subunit p110γ suppresses cardiac contractility and heart rate by reducing intracellular cAMP levels in the ventricular myocardium and sinoatrial node, respectively [25], [28]. This study provides the first genetic evidence that p110α and p110β play pivotal roles in regulating cardiac contractile function by maintaining the T-tubule network. Ablation of both PI3K isoforms in the heart led to T-tubule disorganization and loss that appeared to be due to altered intracellular localization of JP-2. As a consequence, double knockout ventricular myocytes exhibited severe Ca^2+^ handling abnormalities that resulted in heart failure and death.

T-tubule loss or reorganization in ventricular myocytes has been observed in diverse models of heart failure in dogs [7], rats [4], and mice [29]. Decreased physical coupling between Ca_V_1.2 and RyR is thought to be a major cause of altered Ca^2+^ transients and reduced contractility seen in these models [4]. Morphological changes or reduced density of T-tubules have also been seen in ventricular myocytes from humans with different etiologies of chronic heart failure [9], [10]. Rather than being caused by heart failure, changes in the T-tubule network may be an early event in response to cardiac injury. Loss of T-tubules in a pig model of chronic ischemia occurred before the onset of heart failure [30]. In addition, one of us [5] showed that T-tubule loss and remodeling in response to thoracic-aortic banding in rats began before any left ventricular dysfunction was detected and progressed along with the development of overt heart failure. Our finding that 3-week-old dKO mice exhibited T-tubule disarray but not heart failure supports the idea that the ultrastructural abnormalities are the primary result of PI3K deletion, and thus the cause of heart failure. Our results suggest that PI3K signaling is needed to maintain, but not to form, the normal T-tubule network, and with progressive disruption of T-tubules the contractile defect becomes more severe.

The results above suggest that genetic manipulations that directly affect the stability of T-tubules might be expected to cause heart failure. Indeed, whole-body deletion of JP-2 resulted in embryonic lethality [11]. Hearts in JP-2-null embryos showed weak and irregular heartbeats, and cardiac myocytes had abnormal Ca^2+^ transients and were deficient in 12 nm junctions between the peripheral plasma membrane and SR. Acute knockdown of JP-2 in the heart of adult mice also caused impaired cardiac contractility and mortality due to heart failure [14]. Cardiac myocytes from the adult mice exhibited T-tubule disorganization, reduction in the number of junctional membrane complexes, and increased variability in the width of the dyadic cleft. Similar to the acute JP-2 knockdown model, loss of p110α and p110β in our acute PI3K double knockout mice disrupted the structure of an already formed T-tubule network. It is interesting that deletion of JP-2 led to embryonic lethality [11], whereas our PI3K dKO mice died several weeks after birth. A recent study of the postnatal development of cardiac excitation-contraction coupling in the rat showed that RyR localized to the Z line very early in development, while JP-2 localization changed from a peripheral distribution near the plasma membrane in immature myocytes to the jSR membrane as the T-tubule system matured [6]. It is possible that PI3K deletion affects T-tubule structure only after JP-2 and RyR localize to the same compartment. Interestingly, the striated pattern of RyR was disrupted in both chronic and acute PI3K dKO myocytes, suggesting that PI3Ks also regulate membrane organization of the jSR. This could also be dependent on JP-2 localization since this protein was shown to coimmunoprecipitate with RyR [14].

A reduction in the amount of JP-2 protein in the left ventricle was seen in genetic mouse models of hypertrophic or dilated cardiomyopathy [31]. In addition, JP-2 protein was gradually down regulated during the progression from compensated hypertrophy to heart failure in a thoracic-aortic banding rat model of pathological left ventricular afterload [5]. By contrast, we saw mislocalization of JP-2 in myocytes from failing dKO and ind-dKO hearts, but no appreciable loss of JP-2 protein. Interestingly, JP-2 missense mutants found in human hypertrophic cardiomyopathy were reported to mislocalize and perturb Ca^2+^ signaling when expressed in cultured cells of muscle origin [32]. These results suggest that JP-2 loss or mislocalization can lead to cardiac pathology.

PI3K-dependent JP-2 localization might be controlled by the amino-terminal MORN domains [13]. MORN domains in plant phosphatidylinositol 4-phosphate 5-kinases have been shown to bind to phosphoinositides [33], [34], and the cytoplasmic portion of mouse JP-2 containing the MORN repeats was shown to target proteins to the plasma membrane [11]. These results raise the possibility that T-tubule membranes from double PI3K knockout myocytes might contain a reduced amount of PIP_3_ that prevents JP-2 from binding and tethering them to the SR. Future studies will investigate whether the PIP_3_ content of plasma membranes affects the binding of JP-2. JP-2 has also been shown to coimmunoprecipitate with caveolin-3 [31], raising the possibility that protein-protein interactions between JP-2 and PI3Ks might be important for JP-2 localization.

We previously reported that acute deletion of p110α, but not p110β, in myocytes of adult mice caused a reduction in contractility and Ca^2+^ transients [16]. Similar results were seen here in young animals that lacked p110α or p110β during cardiac development. The severity of the phenotype in dKO and ind-dKO mice as compared to the single knockouts could be due to a shared function of the two enzymes in regulating the cellular content of PIP_3_. It has been proposed that p110β synthesizes a basal pool of PIP_3_ that by itself has little effect on Akt, whereas p110α synthesizes a much larger pool in response to growth factor stimulation that is efficiently coupled to Akt phosphorylation [35]. Our observation that insulin-induced Akt phosphorylation was lower in the dKO myocytes than in p110α or p110β single knockout cells is consistent with loss of both PIP_3_ pools. Decreased signaling to Akt in the double knockout myocytes might contribute to the heart failure phenotype. Alternatively, p110α and p110β might have different functions in the heart (e.g., PIP_3_ production by p110α and promotion of autophagy by p110β) that are both required for maintaining T-tubule integrity.

In summary, we have demonstrated that the presence of PI3K p110α and p110β is crucial for maintaining structural integrity of the T-tubule network and normal cardiac function. These results may have implications for the use of broad-spectrum PI3K inhibitors currently being tested in clinical trials for cancer patients [36]. Our results indicate that patients treated with these drugs, especially patients with preexisting heart disease or those with diabetes who may already have reduced PI3K signaling in the heart, should be closely monitored for cardiac side effects. Partial inhibition of PI3K by these drugs could exacerbate the underlying cardiac dysfunction.

## Supporting Information

Figure S1 **PI3K expression and insulin signaling in PI3K knockout hearts.** (**a**) Lysates of myocytes isolated from the indicated groups of mice from the chronic PI3K deletion model were analyzed by western blotting to detect the p110α and p110β PI3K catalytic subunits and the p85 regulatory subunit. GAPDH served as a loading control. (**b**) Mice were fasted overnight, anesthetized and injected through the inferior vena cava with saline or insulin (5 U/kg). Hearts were harvested 5 min later and lysates were analyzed by western blotting with the indicated antibodies. (**c**) Lysates of myocytes isolated from ind-dKO and ind-control mice from the acute PI3K deletion model were analyzed by western blotting to detect the indicated proteins. (**d**) Mice were injected with insulin as described above and heart lysates were analyzed by western blotting with the indicated antibodies.
(TIF)

Figure S2 **Characterization** **of PI3K knockout hearts.** (**a**) Gross morphology of representative hearts from control, α^−/−^, β^−/−^ and dKO mice. Sections were stained with hematoxylin and eosin (H&E). (**b**) Representative high power images of H&E-stained heart sections. (**c**) Real-time RT-PCR analysis of βMHC mRNA levels in control, α^−/−^, β^−/−^ and dKO hearts (*n* = 5 for control and dKO and *n* = 3 for α^−/−^ and β^−/−^; *P<0.05 as compared to control, one-way ANOVA with *post-hoc* Fisher's test). (**d**) Gross morphology of representative hearts from ind-dKO and ind-control mice. Sections were stained with H&E. (**e**) Representative high power images of H&E-stained heart sections.
(TIF)

Figure S3 **Expression of caveolin-3, Ca_V_1.2, RyR and JP-2.** (**a**) Immunoblots of heart microsomal preparations. Samples in the left panel were obtained from 2 pairs of control and dKO hearts. Samples in the right panel were obtained from 2 pairs of ind-control and ind-dKO hearts. (**b**) Confocal microscopy images of isolated ventricular myocytes labeled with antibodies against caveolin-3.
(TIF)

Video S1 **Echocardiogram of a control mouse.**
(AVI)

Video S2 **Echocardiogram of a dKO mouse.**
(AVI)
