# Supplementary figures and images for: PI3Ks Maintain the Structural Integrity of T-Tubules in Cardiac Myocytes
Source: PLoS One. 2011 Sep 2;6(9):e24404. doi: 10.1371/journal.pone.0024404 (PMC3166327; doi:10.1371/journal.pone.0024404)

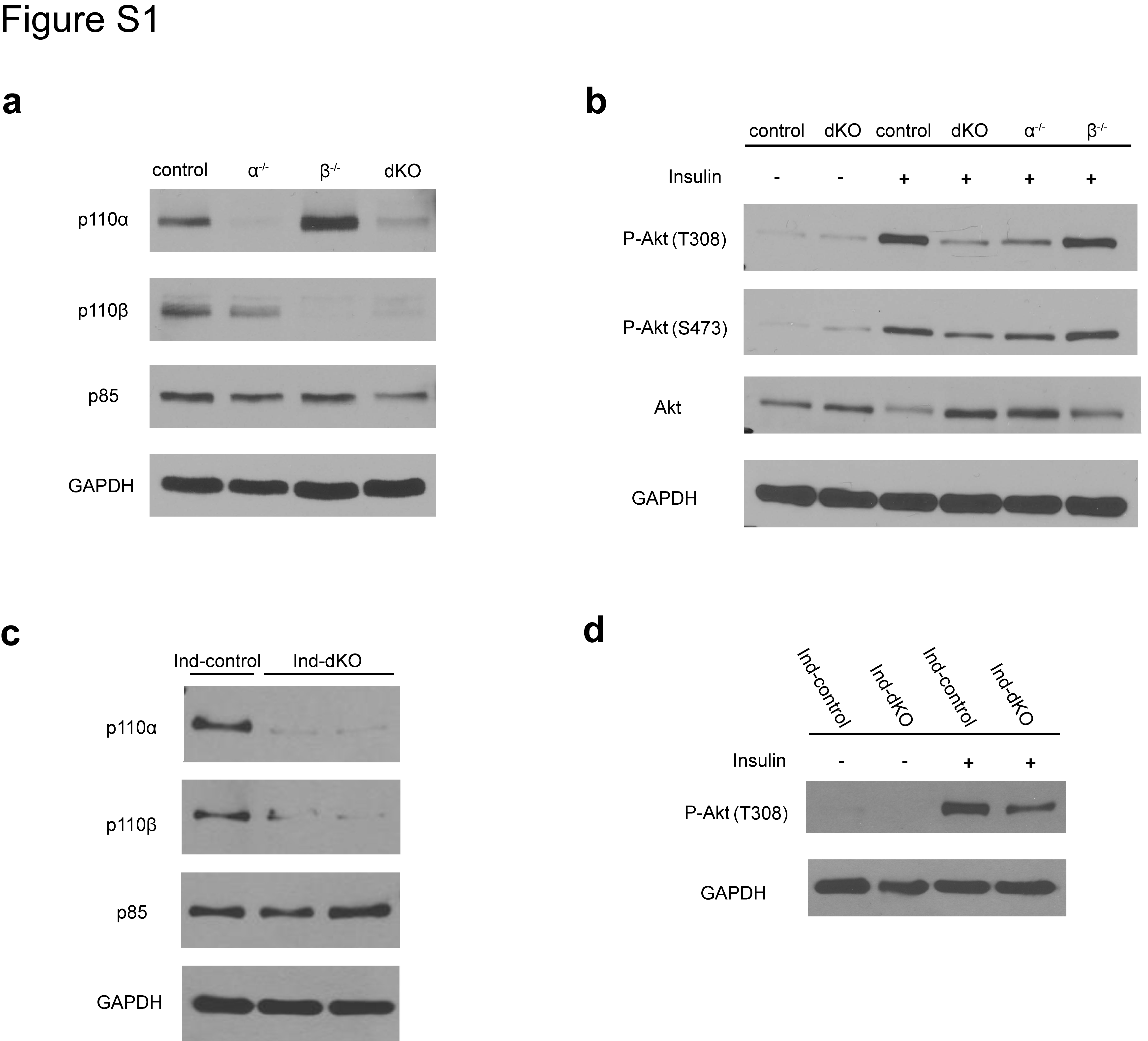

Supplement: Figure S1 — PI3K expression and insulin signaling in PI3K knockout hearts. (a) Lysates of myocytes isolated from the indicated groups of mice from the chronic PI3K deletion model were analyzed by western blotting to detect the p110α and p110β PI3K catalytic subunits and the p85 regulatory subunit. GAPDH served as a loading control. (b) Mice were fasted overnight, anesthetized and injected through the inferior vena cava with saline or insulin (5 U/kg). Hearts were harvested 5 min later and lysates were analyzed by western blotting with the indicated antibodies. (c) Lysates of myocytes isolated from ind-dKO and ind-control mice from the acute PI3K deletion model were analyzed by western blotting to detect the indicated proteins. (d) Mice were injected with insulin as described above and heart lysates were analyzed by western blotting with the indicated antibodies. (TIF) [file pone.0024404.s001.tif]

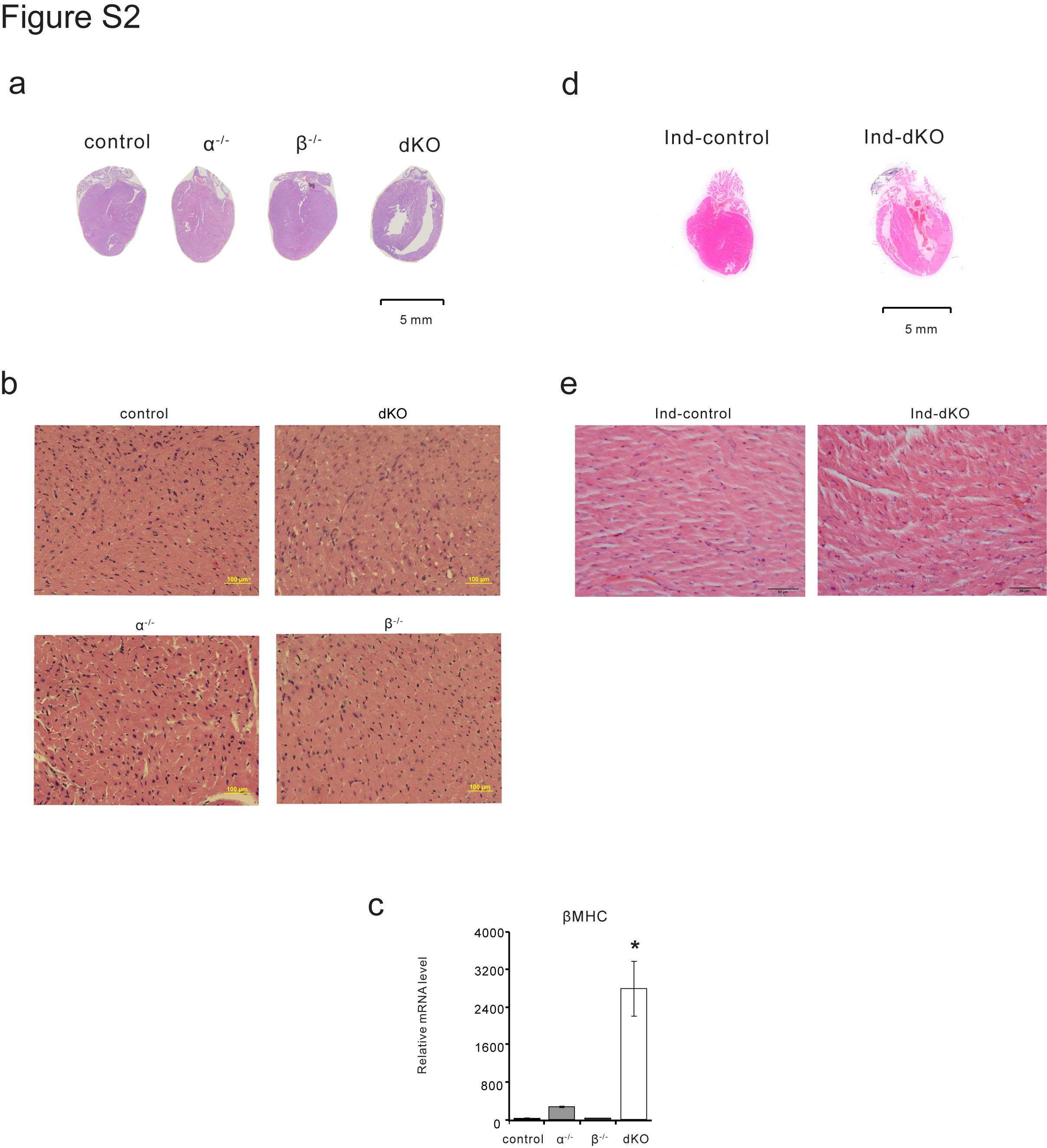

Supplement: Figure S2 — Characterization of PI3K knockout hearts. (a) Gross morphology of representative hearts from control, α−/−, β−/− and dKO mice. Sections were stained with hematoxylin and eosin (H&E). (b) Representative high power images of H&E-stained heart sections. (c) Real-time RT-PCR analysis of βMHC mRNA levels in control, α−/−, β−/− and dKO hearts (n = 5 for control and dKO and n = 3 for α−/− and β−/−; *P<0.05 as compared to control, one-way ANOVA with post-hoc Fisher's test). (d) Gross morphology of representative hearts from ind-dKO and ind-control mice. Sections were stained with H&E. (e) Representative high power images of H&E-stained heart sections. (TIF) [file pone.0024404.s002.tif]

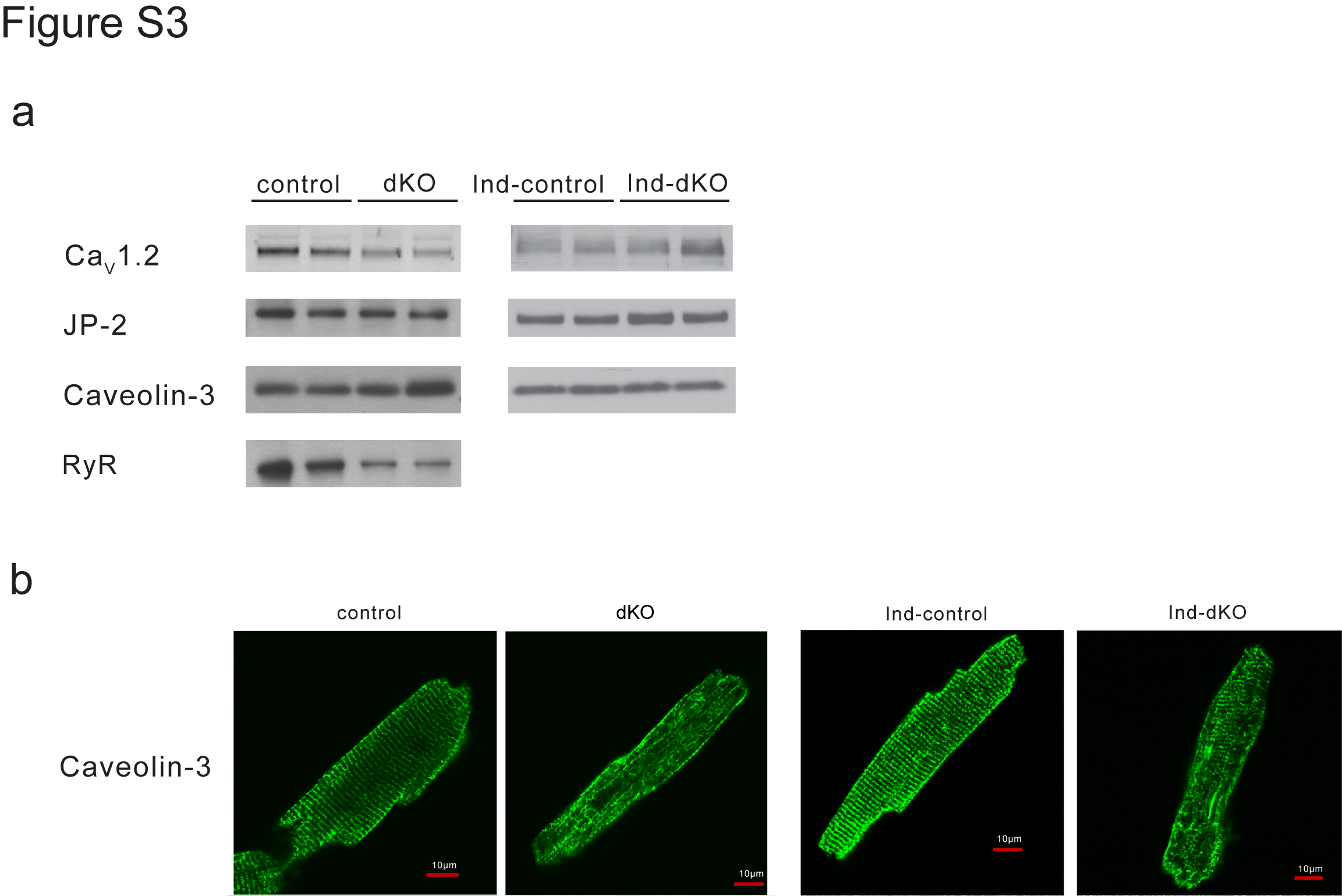

Supplement: Figure S3 — Expression of caveolin-3, CaV1.2, RyR and JP-2. (a) Immunoblots of heart microsomal preparations. Samples in the left panel were obtained from 2 pairs of control and dKO hearts. Samples in the right panel were obtained from 2 pairs of ind-control and ind-dKO hearts. (b) Confocal microscopy images of isolated ventricular myocytes labeled with antibodies against caveolin-3. (TIF) [file pone.0024404.s003.tif]
